# Intravascular Pulmonary Artery Tumour Mimicking Nodal Recurrence and Inadvertently Sampled by EBUS‐TBNA: A Case Report

**DOI:** 10.1002/rcr2.70708

**Published:** 2026-07-30

**Authors:** Shoichiro Harada, Yasuto Ueda, Shinsuke Oda, Shino Arita, Yuri Shibata, Takashi Sumikawa, Yasuyuki Hasegawa, Mizuho Matsushita, Hiroki Chikumi, Akira Yamasaki

**Affiliations:** ^1^ Department of Respiratory Medicine Tottori Prefectural Central Hospital Tottori Japan; ^2^ Department of Pathology Tottori Prefectural Central Hospital Tottori Japan; ^3^ Department of Rheumatology Tottori Prefectural Central Hospital Tottori Japan; ^4^ Division of Medical Oncology and Molecular Respirology, Faculty of Medicine Tottori University Yonago Japan

**Keywords:** diagnostic pitfall, EBUS‐TBNA, intimal sarcoma, pulmonary artery tumour, sarcomatoid transformation

## Abstract

Pulmonary artery tumours are rare and may mimic thromboembolism or nodal recurrence on imaging. We report a woman in her 70s with a history of resected stage IB lung adenocarcinoma who underwent endobronchial ultrasound‐guided transbronchial needle aspiration (EBUS‐TBNA) for a mediastinal lesion suspected to be nodal recurrence. During the second needle pass, synchronous lesion movement and increased bleeding were observed. Histopathology revealed high‐grade sarcomatous cells with weak focal thyroid transcription factor‐1 (TTF‐1) positivity and h‐caldesmon/myogenin expression, unlike the primary adenocarcinoma. Subsequent imaging confirmed that the lesion was intravascular within the pulmonary artery. EBUS‐TBNA may inadvertently sample a pulmonary artery tumour when it mimics nodal recurrence during post‐resection surveillance. Synchronous lesion movement and unexpected bleeding may indicate pulmonary artery puncture. Sarcomatoid transformation of adenocarcinoma and primary intimal sarcoma remained competing diagnoses because tissue was insufficient for molecular confirmation.

## Graphical abstract

An intravascular pulmonary artery tumour mimicked nodal recurrence during post‐resection surveillance and was inadvertently sampled by EBUS‐TBNA. Synchronous lesion movement and increased bleeding were retrospective warning signs of pulmonary artery puncture, while sarcomatoid transformation and primary intimal sarcoma remained unresolved competing diagnoses.

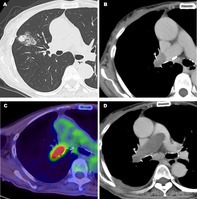

## 1 Introduction

Pulmonary artery tumours are rare and may be indistinguishable from mediastinal nodal disease on imaging. Approximately 20 intravascular pulmonary artery tumours have been diagnosed by endobronchial ultrasound‐guided transbronchial needle aspiration (EBUS‐TBNA) to date, as recently reviewed [1] (11 primary sarcomas, 9 metastatic tumour emboli, 1 inflammatory myofibroblastic tumour); the present case is not included in that review. In most previously reported cases, however, the vascular nature of the lesion was known or suspected before sampling [1]. We report a case in which an intravascular pulmonary artery tumour was inadvertently sampled under the working diagnosis of nodal recurrence, and describe intraprocedural warning signs and the competing differential diagnosis between sarcomatoid transformation and primary intimal sarcoma.

## 2 Case Report

**FIGURE 1:**
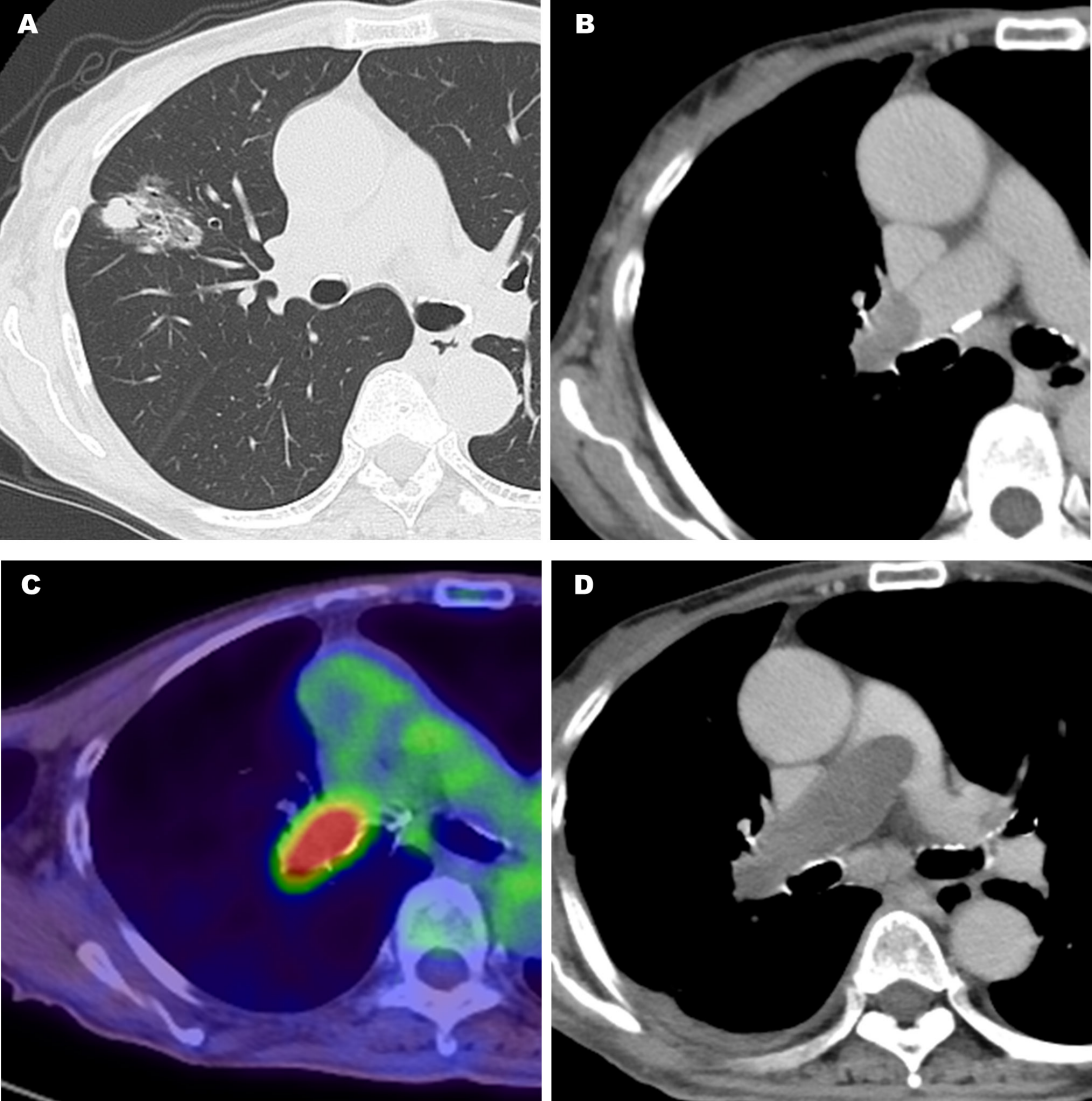
(A) Chest CT at initial presentation showing a 20‐mm nodule in the right upper lobe, consistent with primary lung adenocarcinoma. (B) Follow‐up chest CT 2 years after surgical resection demonstrating a newly appeared mediastinal mass adjacent to the right main pulmonary artery, initially interpreted as lymph node metastasis. (C) PET‐CT showing intense FDG uptake in the mediastinal mass. (D) CT obtained at disease progression confirming intraluminal extension of the tumour from the right main pulmonary artery toward the bifurcation, establishing the intravascular localization of the tumour.

A woman in her 70s underwent right upper lobectomy for stage IB lung adenocarcinoma without driver mutations. Two years later, surveillance computed tomography (CT) revealed a 20 × 25 mm mediastinal lesion adjacent to the right main pulmonary artery (Figure 1A,B). Positron emission tomography‐computed tomography (PET‐CT) showed fluorodeoxyglucose (FDG) uptake with an SUVmax of 8.75 (Figure 1C), and the lesion was presumed to represent nodal recurrence. EBUS‐TBNA was performed with a 22‐gauge needle at station 10R–11 s.

The first pass was uneventful. During the second pass, the lesion moved synchronously with needle manipulation. On needle withdrawal, the mass appeared to be displaced toward the needle tip, transiently revealing an echo‐free space deep to the lesion. Slightly increased bleeding was also observed at the puncture site; the patient was not on antiplatelet or anticoagulant therapy, and preprocedural coagulation studies were normal. These findings were not recognised as clinically significant at the time. Rapid on‐site evaluation confirmed atypical cells, and the procedure was stopped after two passes.

**FIGURE 2:**
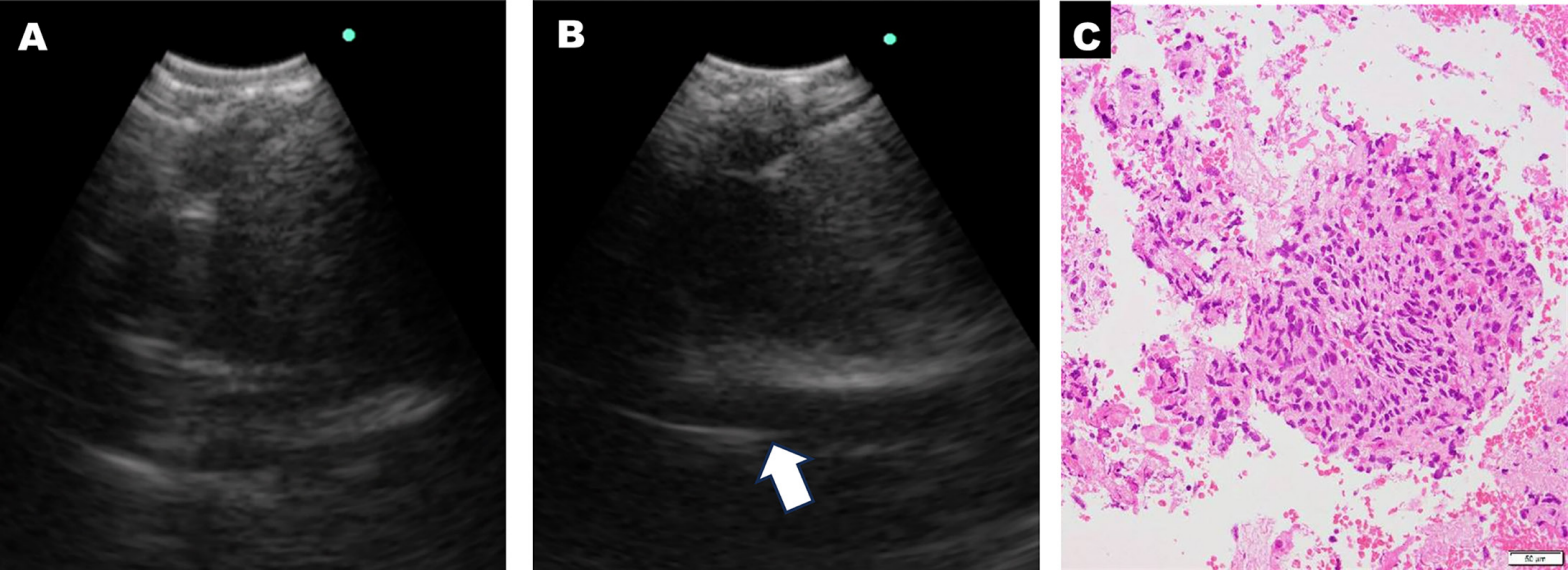
(A) Endobronchial ultrasound (EBUS) image during the first needle pass showing a homogeneous hypoechoic mass at station 10R–11 s with sonographic features consistent with nodal recurrence, the presumed diagnosis at the time. No echo‐free space is visible deep to the mass. (B) EBUS image obtained during needle withdrawal on the second pass. The mass has been displaced superiorly toward the needle tip, revealing a wedge‐shaped echo‐free space deep to the mass (arrow) consistent with the pulmonary artery lumen. This finding suggests that the needle had engaged an intravascular structure, but was not recognised as significant at the time of the procedure. (C) Haematoxylin and eosin staining of the EBUS‐TBNA specimen showing high‐grade sarcomatous cells (original magnification ×200). Scale bar = 50 μm.

**FIGURE 3:**
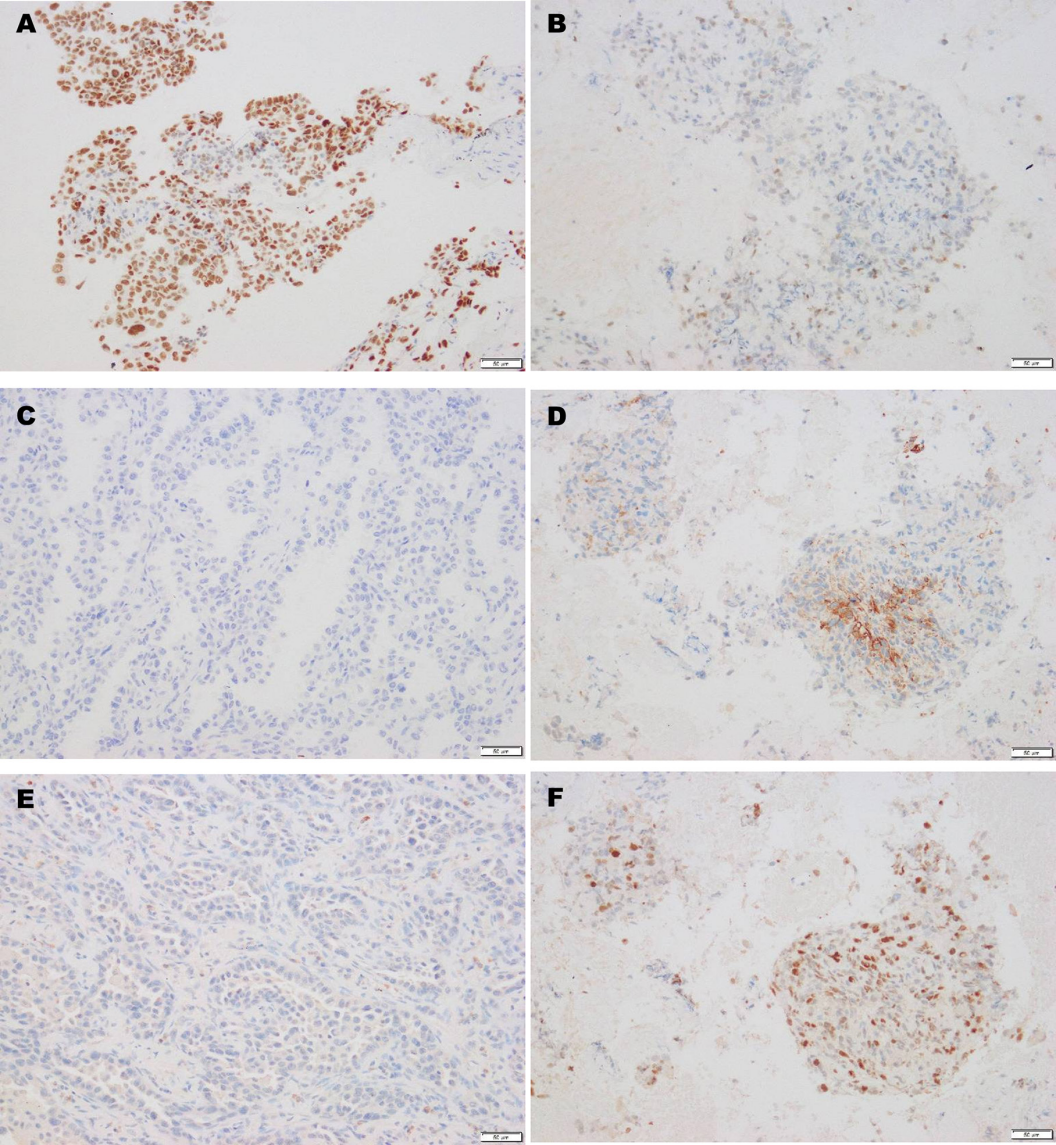
Immunohistochemical comparison between the primary lung adenocarcinoma and the recurrent intrapulmonary artery tumour (original magnification ×200). (A, B) TTF‐1 staining: Strong diffuse positivity in the primary tumour (A) and weak focal positivity in the recurrent lesion (B), consistent with partial loss of epithelial differentiation in the recurrent lesion. (C, D) h‐caldesmon staining: Negative in the primary tumour (C) and positive in the recurrent lesion (D), demonstrating de novo acquisition of smooth muscle differentiation. (E, F) Myogenin staining: Negative in the primary tumour (E) and positive in the recurrent lesion (F), demonstrating de novo acquisition of skeletal muscle differentiation. Scale bars = 50 μm for all panels.

The increased bleeding resolved spontaneously without intervention, and the patient remained hemodynamically stable throughout the procedure and reported no hemoptysis or chest pain afterward. Post‐procedural chest radiography showed no evidence of haemorrhage or pneumothorax. Histopathology showed high‐grade sarcomatous cells in a necrotic background (Figure 2). Immunohistochemistry demonstrated weak focal thyroid transcription factor‐1 (TTF‐1) positivity with newly acquired h‐caldesmon and myogenin expression, in contrast to the strongly TTF‐1‐positive, h‐caldesmon/myogenin‐negative primary tumour (Figure 3; Table 1). Targeted molecular testing detected no driver alterations; tissue‐based comprehensive genomic profiling was precluded by insufficient material; plasma comprehensive genomic profiling detected no actionable alterations.

**TABLE 1:** Immunohistochemical profile of the primary and recurrent tumours.

Marker	Primary tumour	Recurrent lesion
TTF‐1	Strong positive	Weak positive
Napsin A	Positive	Negative
AE1/3	Positive	Negative
CK7	Positive	Negative
h‐caldesmon	Negative	Positive
Myogenin	Negative	Positive
MyoD1	N/A	Positive
αSMA	N/A	Positive
Desmin	N/A	Positive
Vimentin	N/A	Positive
CD34	N/A	Negative
p40	N/A	Negative
CD45/LCA	N/A	Negative
p53	N/A	Negative

Abbreviations: AE1/3, pancytokeratin; CK7, cytokeratin 7; h‐caldesmon, high molecular weight caldesmon; N/A, not assessed; TTF‐1, thyroid transcription factor‐1; αSMA, alpha‐smooth muscle actin.

Doxorubicin monotherapy showed no response after 3 cycles. Follow‐up imaging performed approximately 3 months after the EBUS‐TBNA, at the time of confirmed disease progression following 3 cycles of doxorubicin, demonstrated tumour extension within the right main pulmonary artery toward the bifurcation, establishing the intravascular location retrospectively (Figure 1D). Palliative radiotherapy (40 Gy) was administered, followed by eribulin; the patient has maintained stable disease for 18 months.

## 3 Discussion

We would like to take the opportunity to discuss the learning points from our case.

First, an intravascular pulmonary artery tumour presented as mediastinal nodal recurrence in a patient under post‐resection surveillance, and its vascular nature was not recognised before or during EBUS‐TBNA. Unlike most previously reported cases, in which the vascular nature was known or suspected before sampling [1], this surveillance‐setting scenario may be encountered by any bronchoscopist performing EBUS‐TBNA in patients with a history of thoracic malignancy. The lesion fulfilled standard imaging criteria for nodal recurrence, and no preoperative feature prompted vascular suspicion.

Second, two intraprocedural findings were not recognised in real time. The mass moved synchronously with needle manipulation; during withdrawal, it appeared to be displaced toward the needle tip, transiently revealing an echo‐free space deep to the lesion. Increased bleeding was also observed at the puncture site. In retrospect, both findings were consistent with inadvertent pulmonary artery puncture. Colour Doppler imaging was performed and showed no detectable intraluminal blood flow. In retrospect, this is likely due to near‐complete occlusion of the vessel lumen by the tumour. A limitation of Doppler guidance when applied to severely occluded blood vessels is illustrated. We suggest that synchronous lesion movement or unexplained increased bleeding during EBUS‐TBNA of a paravascular mass should prompt the operator to stop sampling and monitor the patient closely, irrespective of Doppler findings. One procedure‐related death during EBUS‐TBNA of a pulmonary artery sarcoma has been reported [2], underscoring the importance of recognising these warning signs.

Third, the tissue samples demonstrated an immunohistochemical shift. Acquired h‐caldesmon and myogenin were present in the recurrent lesion when previously absent in the primary tumour. TTF‐1 was previously strongly positive and became weakly positive in the recurrent lesion. This immunohistochemical shift indicates divergent mesenchymal differentiation and gives rise to two competing diagnoses: sarcomatoid transformation of the primary adenocarcinoma, supported by the TTF‐1‐positive tumour history and residual TTF‐1 expression [3, 4]; versus primary pulmonary artery intimal sarcoma, supported by the characteristic intraluminal location and the frequent heterologous myogenic differentiation of intimal sarcoma [5]. Notably, the boundary between these categories may itself be ambiguous: the mechanism by which a transformed carcinoma reaches the vessel lumen—direct invasion, hematogenous embolism, or intimal seeding—cannot be established pathologically, and this case may represent either entity.

Fourth, *MDM2* and *CDK4* amplification testing, which would support intimal sarcoma, could not be performed because tissue quantity was exhausted. Plasma profiling was uninformative for this distinction: although no *MDM2* copy‐number gain was identified, the low circulating tumour DNA fraction did not allow exclusion of *MDM2* amplification. As discriminating molecular tests could not be performed, diagnostic resolution between sarcomatoid transformation of the primary adenocarcinoma and primary pulmonary artery intimal sarcoma could not be achieved. PET‐CT showed no other FDG‐avid lesion available for biopsy. A confirmatory rebiopsy of the pulmonary artery lesion could help resolve the histogenesis but has not been pursued, as the patient remains in stable disease; it would be considered if further progression occurs.

In conclusion, intravascular pulmonary artery tumours can mimic nodal recurrence during post‐resection surveillance and may go unrecognized until inadvertently sampled by EBUS‐TBNA. Synchronous lesion movement with the needle and increased bleeding are warning signs of inadvertent pulmonary artery puncture. When prior lung cancer and a sarcomatous intravascular tumour coexist, sarcomatoid transformation and primary intimal sarcoma are competing diagnoses that may remain unresolved when tissue is limited.

## Author Contributions

Shoichiro Harada and Yasuto Ueda conceived the report, interpreted the clinical findings, and drafted the manuscript. Shinsuke Oda performed the pathological diagnosis and immunohistochemical analyses. Shino Arita, Yuri Shibata, Takashi Sumikawa, Yasuyuki Hasegawa, and Mizuho Matsushita contributed to clinical management and data acquisition. Hiroki Chikumi and Akira Yamasaki provided overall supervision and critically revised the manuscript for important intellectual content. All authors read and approved the final manuscript.

## Funding

The authors have nothing to report.

## Consent

The authors declare that written informed consent was obtained for the publication of this manuscript and accompanying images using the consent form provided by the Journal.

## Conflicts of Interest

The authors declare no conflicts of interest.

## Data Availability

The data that support the findings of this study are available on request from the corresponding author. The data are not publicly available due to privacy or ethical restrictions.
